# Identifying Barriers to and Opportunities for Telehealth Implementation Amidst the COVID-19 Pandemic by Using a Human Factors Approach: A Leap Into the Future of Health Care Delivery?

**DOI:** 10.2196/24860

**Published:** 2021-04-09

**Authors:** Tianyi Zhang, Jarrod Mosier, Vignesh Subbian

**Affiliations:** 1 Department of Systems and Industrial Engineering College of Engineering The University of Arizona Tucson, AZ United States; 2 Department of Biomedical Engineering The University of Arizona Tucson, AZ United States; 3 Department of Medicine Division of Pulmonary, Allergy, Critical Care and Sleep University of Arizona College of Medicine Tucson, AZ United States; 4 Adult ECMO Service Banner - University Medical Center Tucson Tucson, AZ United States

**Keywords:** telehealth, healthcare system, COVID-19, human factors, implementation, SEIPS

## Abstract

The extensive uptake of telehealth has considerably transformed health care delivery since the beginning of the COVID-19 pandemic and has imposed tremendous challenges to its large-scale implementation and adaptation. Given the shift in paradigm from telehealth as an alternative mechanism of care delivery to telehealth as an integral part of the health system, it is imperative to take a systematic approach to identifying barriers to, opportunities for, and the overall impact of telehealth implementation amidst the current pandemic. In this work, we apply a human factors framework, the Systems Engineering Initiative for Patient Safety model, to guide our holistic analysis and discussion of telehealth implementation, encompassing the health care work system, care processes, and outcomes.

## Introduction

COVID-19, caused by the novel coronavirus SARS-CoV-2, has swept across the globe since its emergence in late 2019. The rapid spread of SARS-CoV-2 imposed an excessive burden on health care systems, with nearly 326.7 per 100,000 people in the USA requiring hospitalization through the end of 2020 [1]. The extensive adoption of telehealth approaches, as part of protective measures and to promote the overall safety of patients and health care workers, has manifested in various essential components of care delivery. The surge in the adoption of telehealth, however, imposes significant challenges to the health care system, as it has disrupted the balance of the health care work system, thus highlighting the importance of exploring the barriers to and impact of the pandemic-driven, large-scale uptake of telehealth technologies.

The health care system is particularly vulnerable to novel and highly infectious agents such as SARS-CoV-2 because of the exponentially increased demand of health care resources [2], including ventilators and personal protective equipment (PPE), and the high risk of infection among care providers through aerosol transmission during clinical care [3], especially by asymptomatic carriers. The most widely adopted strategy among the general public to lengthen the doubling time of the virus and reduce the basic reproduction number, R_0_, involved social distancing to attenuate the proximity and duration of contact with potentially infected individuals. In clinical settings, telehealth solutions have emerged as an effective tool for health care systems to deliver care to patients while minimizing safety risks to both patients and providers by maintaining social distancing.

The Systems Engineering Initiative for Patient Safety (SEIPS) model [4,5] provides a useful framework for analyzing the widespread adoption of telehealth in response to the COVID-19 crisis. This model allows for a comprehensive and proactive assessment of telehealth implementation in the longer term, beyond the pandemic [6]. Previous studies [7-9] discussing barriers to or the impact of telehealth implementation either overlooked patients’ perspectives or focused more on certain components of the health care system. Here, we demonstrate the application of the SEIPS model to guide the assessment of the barriers to and impact of telehealth on health care *systems*, *processes*, and *outcomes* during the ongoing crisis. According to the SEIPS model, the health care work system includes the following components: *person, technologies, environment, tasks,* and *organization*.

- The *person* component considers education, knowledge, motivation, and physical and psychological characteristics, as it relates to both patients and health care providers.
- The *technologies* component involves all devices and information systems that are used to deliver care.
- The *environment* component consists of the workstation design, layout, noise, and any existential environmental factors.
- The *tasks* component discusses the content, participation, and demands of the job.
- Finally, the *organization* component emphasizes teamwork, coordination, collaboration, communication, and organizational culture [4].

The SEIPS model also emphasizes that the analysis of processes and outcomes should be based at both the individual level (ie, patients and health care workers) and the organizational level. For example, telehealth can reduce the burden of *environment* infection due to COVID-19, which is an essential process to health care organizations but not necessarily a direct part of patients’ care processes. In this work, we use the SEIPS model to discuss barriers related to and impacts of telehealth implementation amidst the COVID-19 pandemic (Table 1). Given that there are few measures to assess outcomes of telehealth during the pandemic, we will also discuss and propose measures that could be helpful in guiding the assessment as well as future scalability and efficacy studies.

**Table 1:** Assessment of barriers related to and impact of telehealth implementation during the COVID-19 pandemic by using the Systems Engineering Initiative for Patient Safety (SEIPS) model.

Domain and components		Impact		Issues
**Telehealth-enabled work system**				
	Person as patients	Increased acceptance of telehealth due to convenience	Insufficient and variable levels of digital literacy among the patient population Widening of health care disparities [10-12]	
	Person as providers	Increased motivation Alleviation of workforce shortage due to the quarantine	Mental or physical challenges due to the imperative and wide adoption of telehealth	
	Technologies and tools	Enhanced patient and health care worker safety Conserve PPE^a^	Telehealth may be disruptive and not user-friendly	
	Environment	Highlighted the suboptimal and complex environment for telehealth uptake	Insufficient communication infrastructure The environment where patients interact with telehealth technology may be suboptimal	
	Tasks for patients	Safer and potentially quicker access to care	Systemic, informational, procedural gap that patients need to fill in	
	Tasks for providers	Clinical and nonclinical services can be safely continued via telehealth	Challenges in adapting to changes in job content and demands	
	Organization	Formulation of new teams Maximizing the utilization of existing resources to deal with the pandemic	Dynamic changes to teamwork Reallocation of accountability and responsibility Redistribution of labor, equipment, information, and funding resources	
**Telehealth-enabled processes**				
	Care Processes	Wide application of forward-triage, tele-intake, and tele-ICU^b^ Increase in web-based visits replacing in-person visits and mixed processes (ie, some in-person visits and some televisits)	Time management is more challenging (eg, a busy lobby makes it easier to accept the physician being late as opposed to being at home waiting alone in the virtual lobby) Telehealth may not lead to a shorter overall time spent in the care system	
	Other processes	Reduced demand of other processes that support care processes (eg, reduced environment disinfection needs due to the fewer in-person visits)	Information flow may be more fragmented	
**Telehealth outcomes**				
	Patients’ outcomes	Unclear	Lack of measures for patient safety and quality of care evaluation	
	Care providers’ and organizational outcomes	Unclear	Lack of measures for assessing care providers’ mental and physical health affected by the surging use of telehealth during the COVID-19 pandemic Organizational outcome related to the pandemic-driven, large-scale uptake of telehealth needs more attention	

^a^PPE: personal protective equipment.

^b^ICU: intensive care unit.

## Discussion

### Person

During various stages of the COVID-19 pandemic, many health care providers were quarantined after potential exposure to or confirmed infection with the virus, resulting in a limited workforce and a reduced health care system capacity. Telehealth can facilitate the rearrangement and reassignment of the workforce and maintain the capacity by allowing quarantined health care providers to continue their work without compromising the health care system’s safety. Moreover, care facilities that lack telemedicine programs can outsource part of their services to entities with well-established telemedicine programs to meet these goals [13].

Despite the claimed benefits of efficiency and convenience offered by telehealth, not all health care providers have been satisfied with the telehealth options available, even before the COVID-19 pandemic. Preliminary reports from the early phases of the pandemic [8] suggests that health care providers’ unwillingness is one of the barriers to telehealth implementation in practice. However, this position does not fully consider latent factors such as technological and administrative issues that lead to the active failure (ie, care providers’ unwillingness). Furthermore, there is significant variability in telehealth education and training among clinicians, leading to varying levels of acceptance and uptake. The surging health care demands during the pandemic has forced health care workers to adopt telehealth predominantly for safety reasons. Further research is needed to better understand how the adoption of telehealth demanded by new care delivery protocols may affect health care providers’ physical and mental workload.

Patients, on the other hand, are also profoundly influenced by the imperative uptake of telehealth since the beginning of the pandemic. Recent studies have shown that telehealth approaches such as remote video visits in a variety of care delivery contexts is acceptable to patients [14,15]. For example, some patients perceive primary care video visits as convenient and efficient because they can stay in their home environments while seeing care providers; however, they are still concerned about privacy issues [14]. A 2009 systematic review [16] and a recent study [17] have both identified that factors such as human-technology interaction (ie, user experience and usefulness), environment (ie, the context where patients would use telehealth), and patient demographics (ie, socioeconomic status) could influence their acceptance of telehealth. Although the pandemic may have potentially increased patients’ subjective acceptance of telehealth, objective barriers still hinder a higher acceptance among patients. For instance, some patients may not have access to technology that enables telehealth, have poor internet connectivity, or face technical challenges in navigating telehealth systems [14,18]. These barriers are particularly encountered by vulnerable populations that need most medical attention during the COVID-19 pandemic. In general, although the public health crisis may improve the overall uptake, penetration, and implementation of telehealth among all populations, it may also intensify health inequities [10-12].

### Technologies and Tools

Prior to the COVID-19 pandemic, telehealth was regarded as an alternative form of care delivery to in-person care. It was considered ancillary because telehealth was not widely possible until the widespread prevalence of smartphones [19,20]. Today, telehealth can be realized through a variety of communication modalities depending on institutional and regulatory guidance, such as phone calls, text messaging, email, patient portal, or licensed third-party software, and most of them can be accomplished via smartphones.

A recent study pointed out that the adoption of telehealth can conserve PPE [21], which was severely limited in supply during the pandemic. The demand for acute care surged and quickly surpassed the system capacity in many US states. Therefore, by performing select services via telemedicine, hospitals and clinics can conserve PPE and extend the time to peak capacity. Even high-volume emergency departments can preserve PPE and safety by performing medical screening exams remotely for patients with suspected COVID-19 [21].

Despite the benefits of telehealth uptake, we cannot assume it would work well within the current health care system. In fact, the telehealth system is deemed as disruptive and not user-friendly by many clinicians [8]. For instance, in large health systems in urban Southwest Arizona, telehealth tools were made available during the pandemic, yet many were impractical or nonviable solutions. Contrary to the report that claimed clinicians’ unwillingness of adopting telehealth [8], clinicians were positive about telehealth and eager for its uptake to continue serving their patients during statewide mandatory stay-at-home orders, but they were also frustrated at the obstacles to its implementation. We believe that a redesign of the telehealth system is urgently required and is fundamental to higher levels of acceptance and satisfaction among users, including both patients and providers.

### Environment

Despite the convenience that telehealth can provide, the lack of infrastructure and insufficient technical capability may limit providers’ and patients’ use and acceptance of telehealth. Although the majority of the United States has access to 4G or faster networks, many remote and rural regions still lag behind in terms of internet coverage. A report from the American Hospital Association shows that 34 million Americans do not have access to satisfactory broadband [22]. The existing Federal Communication Commission program that supports the expansion of broadband is criticized as cumbersome and insufficient to fill the financial gap of increasing broadband access in rural areas [22]. Previous studies have found that telehealth is an effective tool to treat a large group of patients in disaster response [23] and that Wi-Fi and cellular service are key to the successful implementation of telemedicine [19]. The poor coverage may limit, for example, the quality of video conferences between patients and health care workers, or even between health care workers from remote areas. Even in developed health care facilities or regions, the communication demands during the COVID-19 pandemic may still impose a heavy load on the hospitals’ network, thus hindering telehealth capabilities and requiring immediate technical attention. One solution to improve telehealth use could be to deliver some data using 4G as well as 2G and 3G networks [24], which could ease the burden on the network.

The COVID-19 pandemic may have also changed the environment wherein the patient usually uses telehealth. The environment in which patients interact with telehealth technology may be suboptimal. The shelter-in-place orders compelled people to stay in their residential living spaces. The lighting, noise level, and airflow in residential living spaces may not be ideal for medical consultation via telemedicine. For example, childcare and at-home responsibilities may interfere with the interaction with providers via telemedicine, especially regarding sensitive issues. Such environmental factors are less often explored by studies but are still demanded important for satisfactory telehealth use [16].

### Tasks

Telehealth can facilitate the delivery of clinical and nonclinical services [25], both of which are essential during the COVID-19 pandemic. Telehealth-enabled clinical services usually consist of live, video-based patient visits, store-and-forward consultations (eg, patients measure their body temperature at home and care providers evaluate the information in the remote setting), remote monitoring (eg, electronic intensive care unit [e-ICU] [13]), messages sent through phone or a patient portal, and phone calls [25,26].

The widespread adoption of telehealth to deliver care during the COVID-19 pandemic has changed the care delivery protocols [27]. Patients are expected to collaboratively fill systemic (eg, navigating an unfamiliar method of accessing care), informational (eg, primary care physicians cannot visually examine the patients if the consultation is realized via messages or phone calls, and their diagnosis would only be based on verbal descriptions, which is filled in by patients), or procedural (eg, recording their own vital signs prior to video consultation or store-and-forward consultation) gaps. As patients are required to take more responsibility with telehealth, they could feel overwhelmed and disoriented about navigating the rapidly changing system of care. Thus, it is vital to ensure the design of a telehealth-mediated health care system is centered on patients’ needs and experiences.

Current telehealth practices have also disrupted care providers’ workflow and work content [28,29]. In a report describing a Veterans Affairs physician’s day of tele-interacting with their patients in the era of the COVID-19 pandemic, the provider was frustrated that telehealth does not allow them to quickly grasp important peripheral information of patients, such as their comportment and facial expressions, which in-person visits allow for [29]. The peripheral information is essential to providers particularly for older patients or those with various underlying health conditions, that is, groups that are the most vulnerable to COVID-19. This is not an isolated example, and there are more care providers affected that need to make the quick switch to telemedicine to be compliant with the new care policies during the COVID-19 pandemic. This introduces extreme stress into the health care system, especially when additional training is often necessary. Above all, telehealth as a protective method for health care workers during the pandemic is mostly leading to the positive outcomes, but its negative effects should also be addressed. For example, it is advised that establishing routine tele–follow-up communications is one way to help attenuate the negative effects of disruption, as it keeps clinicians informed of their care decision results and enables them to maintain the continuity of care [30].

### Organization

During the COVID-19 pandemic, many facilities were required to form new teams that were specifically designated to tackle all COVID-19–related activities, including care coordination. For instance, an academic medical center in San Diego formed an “Ambulatory COVID Team (ACT)” consisting of seven care providers, including physicians, nurses, epidemiology experts, and administrative officers [30]. Furthermore, the guidelines for crisis standards of care at short-term inpatient acute care facilities, published by individual states, urges all health care facilities to assemble a committee that is designated to review and implement guidance during the COVID-19 crisis [31]. Such new teams and committees would change the teamwork dynamic within their health care system.

Considering e-ICU programs as an example of telehealth, several programs provided services to allow physicians and nurses to remotely monitor patients in ICUs across several hospitals [13]. The services from these institutions can reduce the burden on and needs of the health care workforce in local hospitals and may be more cost-effective than the traditional ICU setting. The challenges remain in terms of how remote clinicians communicate, collaborate, and coordinate with onsite health care workers to deliver timely and necessary care to patients that are compliant with the safety standards. The overall management and organization of ICU may require further analysis as to who should be accountable for patient safety.

In addition to the numerous changes telehealth could possibly contribute to the teamwork dynamics, it could also play a role in resource reallocation within the system. Resources in health systems usually include labor resources (ie, a variety of care providers and administrative staff), equipment resources (ie, ventilator, PPE, and computers), information resources (ie, patient information), and funding resources (ie, payment and reimbursement).

Telehealth can conserve valuable labor resources and maximize the use of available human resources by (1) protecting health care providers from potential exposure to COVID-19; (2) allowing health care providers with suspected exposure to COVID-19 to continue working, who may otherwise have to be self-isolated [13]; (3) integrating labor resources across different systems (eg, e-ICU can reduce the onsite labor resource requirement by using centralized patient monitoring [13]). Ideally, telemedicine can help out-of-state providers to fill in the local shortage of health care workforce economically and promptly [19]; however, state-based physician licensure can hinder the use of telemedicine to coordinate the cross-state response to a natural disaster such as COVID-19 pandemic [26]. Fortunately, the pandemic has effected changes to several policies, as the federal and state governments have modified or waived certain policies to facilitate the broad application of telehealth [30].

Telehealth use was mostly restricted to patients living in remote areas or staying in the health care facilities [22]; therefore, most patients, even if they wanted telehealth services, did not have many options to do so, given the billing and insurance coverage concerns associated with its use. One report listed reimbursement problems as one of the barriers to the use of telehealth [8]. In the United States, a recent survey revealed that the District of Columbia and 42 out of 50 states have enacted some telehealth commercial insurance coverage policy [32], and only 35 states together with the District of Columbia have some sort of parity law [22], which direct insurance providers to cover telehealth services the same way as they would cover in-person care services. However, in many states, the details of reimbursement policy of telehealth are still vague as payment parity is unclear as well. The payment parity means that telehealth services should be reimbursed to the same extent as how traditional in-person services are reimbursed. Given the special circumstances of the COVID-19 pandemic, wherein many fees are subsidized or waived, parity payment is not a significant concern at this stage but telehealth insurance coverage is still a dominating issue. As urban dwellers are more in need of telehealth due to the higher likelihood of spread of the virus within areas of relatively higher population density [30], the traditional telehealth reimbursement policies would pose as a barrier to applying telehealth during the COVID-19 pandemic. In 2018, Duffy and Lee [20] suggested that providers need to actively redesign the care models and that the payment system will evolve along with it. Fortunately, this is no longer the case. Current Medicare coverage in the United States has removed the rural and site limitations and allows patients residing in any location to get covered for their telehealth use [33].

The pandemic-driven telehealth uptake also heightened the information flow problem more than ever before. A COVID-19 care management pathway enabled by telehealth can connect many health care entities for triaging, screening, and treatment through telehealth or onsite outpatient visits, specimen collection (onsite or drive-through), clinical testing laboratories, follow-up with primary care or appropriate care providers, and inpatient care [34]. Information flow among these entities is often fragmented due to a plethora of regulations and laws such as the Health Information Technology for Economic and Clinical Health Act and Health Insurance Portability and Accountability Act (HIPAA) [34]. The multiple overlapping federal and state laws that intentionally protect health information located in different information systems now unsurprisingly also make it onerous for care providers and patients to use telehealth to exchange COVID-19–related information [35] Therefore, the difficulty of integrating patient health information across entities needs to be addressed for effective telehealth services. Improving interoperability between various information systems and enhancing electronic health record as a one-stop information hub may be one potential solution [34].

### Processes

The clinical care processes of COVID-19 typically consist of four stages: screening, testing, treatment, and recovery. During the screening stage, forward triage is deemed as an important practice to relieve the intake pressure on the health care facility’s front end [13]. Ideally, forward triage is done through telehealth where initial risk assessment and patient counseling are conducted remotely [34]. This would give patients quicker access to care while keeping low-risk patients away from the overwhelmed health care system and reducing unnecessary exposure for patients and care providers. In addition to the forward triage, tele-intake is also a good approach to reduce exposure risks for some in-person visits if deemed necessary [13]. It is noteworthy that the use of telehealth may not reduce the overall time that patients spend in the health care system, starting from the initial contact with the health care system until their last contact, because telehealth may not address the bottlenecks of the entire treatment management process that cause delays. For example, forward triage can reduce patients’ time of accessing care, but they may still need to wait for a hospital bed during their actual in-person visit. One study found that tele-intake can increase the rate of leaving without treatment completion and that tele-intake only functions best when the health care system capacity levels up accordingly [36].

While telehealth had manifested its potential in allowing patients quicker access to care, it also imposed a higher requirement for care providers’ time management. A traditional busy lobby usually made it easier to accept if the physicians were late to the appointments; however, patients waiting alone on a web-based platform and not being able to see the bustle on the side of care providers could make the care experience less patient-centered. Hence, an ideal telehealth system design would allow care providers to better engage patients and improve their care experience before and after the televisit.

In terms of other processes that support care processes, telehealth may exhibit different effects. For example, with fewer in-person visits, the stress and demand of repetitive environment disinfection could be relatively relieved. However, telehealth could also impose extra challenges of integrating, maintaining, and transferring of patient information. 

### Outcomes

Regulation and policy changes that have come into effect during the pandemic may be perceived as the driving force for the large-scale uptake of telehealth. However, for longer-term sustainability, performance-based outcome metrics are needed to assess the impact of telehealth on health systems. A few studies have made initial attempts to apply existing performance metrics to assess telehealth implementation [36,37]. The outcome measures we propose (Table 2) can help guide future work in optimizing and scaling telehealth implementation.

**Table 2:** Potential outcome measures of telehealth-enabled care.

Outcome level and dimension		Potential outcome measures
**Patient outcome**		
	Patient safety	Diagnostic errors (compared to in-person visits) Hospitalization rate ICU^a^ admission rate Intubation rate Mortality rate (general and ICU) Readmission rate Health care–associated infections
	Care quality	Left without being seen Door-to-provider and door-to-disposition times Left without treatment complete Left against medical advice Left without treatment [36]
**Employee outcome**		
	Work safety	Work-associated infections PPE^b^ sufficiency
	Work quality	Work stress and clinician burnout Work efficiency
Organizational outcome		Staff turnover rate Policy implementation performance Finance health index (before and after the COVID-19 pandemic)

^a^ICU: intensive care unit.

^b^PPE: personal protective equipment.

## Conclusions

The COVID-19 pandemic has thrust telehealth solutions into the front line of health care despite significant barriers to its effective implementation and optimization. There are significant benefits to utilizing telehealth, namely providing enhanced safety options for patients and health care providers during the pandemic and introducing the potential to enhance efficiency and convenience in the future. However, challenges with telehealth implementation arising in different domains of health care work system and processes that potentiate disruption to care delivery, worsen disparities in health care, and provoke changes from different levels within the health care industry, still need to be addressed. Future efforts should therefore address these barriers to implementation by redesigning telehealth solutions via a systematic approach such that health care systems can mitigate the negative effects of telehealth and seamlessly realize the benefits and enhanced safety that telehealth provides.

## Abbreviations

e-ICU: electronic intensive care unit
ICU: intensive care unit
PPE: personal protective equipment
SEIPS: Systems Engineering Initiative for Patient Safety Model
